# Population Structure, Genetic Diversity, and Conservation Strategies of a Commercially Important Sleeper Fish, *Odontobutis potamophilus* (Gobiiformes: Odontobutidae) Based on Gene-Capture Data

**DOI:** 10.3389/fgene.2022.843848

**Published:** 2022-05-24

**Authors:** Yun Hu, Hongjie Li, Jianhong Xia, Chenhong Li

**Affiliations:** ^1^ Shanghai Universities Key Laboratory of Marine Animal Taxonomy and Evolution, Shanghai Ocean University, Shanghai, China; ^2^ Engineering Research Center of Environmental DNA and Ecological Water Health Assessment, Shanghai Ocean University, Shanghai, China; ^3^ Shanghai Natural History Museum, Branch of the Shanghai Science and Technology Museum, Shanghai, China

**Keywords:** *Odontobutis potamophilus*, population structure, conservation, genetic diversity, gene capture, genetic resource

## Abstract

*Odontobutis potamophilus* is a popular food fish in China, distributed mainly in the middle and lower reaches of the Yangtze River, where it is a famous delicacy and a newly focused species for aquaculture. The wild populations of *O. potamophilus* are facing the problem of overfishing and habitat degradation. Therefore, it is very necessary to investigate and protect the wild populations of *O. potamophilus*. In this study, 72 fish were sampled from 18 different sites over its distribution range. Nuclear sequence data of 4,267 loci were collected using a gene-capture method. Phylogenetic reconstruction revealed that there were three major clades: Oujiang clade (OJ), Qiantang and lower Yangtze clade (QY), and middle Yangtze clade (MY). The discriminant analysis of principal components (DAPC) and a STRUCTURE analysis confirmed that there are three major groups within *O. potamophilus*. A fastsimcoal2 analysis corroborated the population history and suggested that there was discernible gene flow among these three groups, especially between QY and MY. Estimated pairwise *F*_*ST*_ suggested that Linhai (LH) and Shexian (SX) populations were the most divergent pair (*F*_*ST*_ = 0.7077). Taking the nucleotide diversity, population divergence, and admixture status altogether into consideration, we recommend that the LH, Gaoyou (GY) and Chaohu (CH) populations could be protected as the preferred resource for breeding projects. According to the results of genetic analyses, all populations of *O. potamophilus* should be protected due to low genetic diversity.

## Introduction

Genus *Odontobutis* contains a group of freshwater sleepers endemic to China, Japan, and the Korea Peninsula (Wu, 2008). There are eight species within this genus, *O. haifengensis*, *O. hikimius*, *O. interrupta*, *O. obscura*, *O. platycephala*, *O. potamophilus*, *O. sinensis*, and *O. yaluensis* (Froese and Daniel, 2014). In China, the two most interested species are *O. potamophilus* and *O. sinensis* because of their high economic value, so most of the research was focused on the reproduction and breeding of these two species (Liu et al., 2008; Zhao et al., 2009). Li et al. (2017) compared the average weight and absolute fecundity between *O. potamophilus* and *O. sinensis* found from Jiangxi Province of China, and found that *O. potamophilus* was more suitable for artificial propagation and aquaculture development than *O. sinensis*.

*Odontobutis potamophilus* is an economically important fish endemic to China. It is mainly distributed in the middle and lower reaches of the Yangtze River, as well as the Huaihe River, the Qiantang River, and the Oujiang River basins (Wu, 2008). *Odontobutis potamophilus* is small carnivorous fish that lives at the bottom of lakes, rivers, and ditches. It prefers to inhabit shallow waters where sand, weeds, and gravel are mixed (Wu, 2008). This fish has high meat-to-body weight ratio (64.45%) (Hu et al., 2012), fine meat quality, and a delicious flavor, but its natural yield is low. Therefore, *O. potamophilus* has become an attractive species for aquaculture in recent years (Wang et al., 2016).

With the increasing market demand, the breeding population of *O. potamophilus* has been in short supply (Zhang et al., 2019). In addition to being caught directly for the market, wild *O. potamophilus* has also been caught for the cultivation of cultured parent fish (Zhang et al., 2012). Driven by the rising price (USD 25 per Kg); the fishing enthusiasm of fishermen is unprecedented (Zhang et al., 2012; Zhang et al., 2019). With the increasing pollution of natural waters, the living environment of *O. potamophilus* was deteriorating and the spawning environment was destroyed repeatedly (Zhu et al., 2014; Wang et al., 2018). As a result of many factors, the natural resources of *O. potamophilus* were increasingly exhausted and the populations declined seriously (Zhao and Sheng, 2011). In the 2017 IUCN Red List, *O. potamophilus* was rated as an information deficient (Data Deficient, DD) species (Osipova et al., 2017). Analyzing the population structure of *O. potamophilus* is the first step to conserver its wild genetic diversity. Wang et al. (2016) hybridized three different geographical populations of *O. potamophilus* collected from Jiande, Zhejiang Province, Dangtu, Anhui Province, and Taihu, Jiangsu Province. They found that the growth rate of F1 hybrids between different populations was all higher than self-propagated within the same population. Therefore, investigating the genetic diversity of different populations also can provide more favorable germplasm resources for aquaculture. There is an urgent need for the estimation of genetic resources of *O. potamophilus*.

At present, most genetic studies on *O. potamophilus* were based on mitochondrial loci (Xu et al., 2015; Zhang et al., 2019), but see Zhu et al. (2014) for developing microsatellite markers for this species and population study covering its partial distribution. They discribed the genetic diversity of *O. potamophilus* in five populaitons, and suggested that *O. potamophilus* in those areas can be used for breeding and conservation programs (Zhu et al., 2014). Hou et al. (2014) show that genetic diversity was low (overall Pi = 0.04028) in *O. potamophilus*, and there was a high degree of differentiation (average *F*_*ST*_ = 0.92911) among populations, absenting gene flow between populations. However, in the studies of Hou et al. (2014) and Zhu et al. (2014), not the whole distribution range of the species was covered. Some populations of *O. potamophilus* with low genetic diversity were found (Hou et al., 2014; Zhu et al., 2014). One of the reasons for low genetic diversity could be inbreeding. Low genetic diversity will cause serious ecological consequences, such as reducing the ability of the population to survive in a stressful or changing environment, and eventually extinction (Hughes et al., 2008). Therefore, it is necessary to protect *O. potamophilus* with low genetic diversity. Most populations of the *O. potamophilus* from different geographic regions have not expanded (Hou et al., 2014). It is important to use multiple nuclear gene loci to evaluate the genetic resource of *O. potamophilus* in its whole distribution range.

Various methods have been applied for collecting thousands of nuclear loci for population studies, such as genotyping-by-sequencing (GBS), restriction site associated DNA sequencing (RAD Sequencing) (Baird et al., 2008; Elshire et al., 2011). Another strategy of reduced representative sequencing is through gene capture. Jiang et al. (2019) developed a suite of 4.434 single-copy nuclear gene markers for ray-finned fishes. Those markers can be used to study both phylogenomics and population genomics of ray-finned fishes, and those have been successfully tested in *Odontobutis* species (Li et al., 2018; Jiang et al., 2019).

In this study, we collected 72 fish from 18 different sites of the Huaihe River, the Yangtze River, the Qiantang River, and the Oujiang River basins. We applied a target-gene enrichment approach (Li et al., 2013) and the next generation sequencing method to obtain a large amount of nuclear gene data, and carried out on estimation of the genetic diversity and population structure of *O. potamophilus*. The results of this research can provide a reference for the selective breeding programs of *O. potamophilus* with high genetic diversity or populations with low genetic diversity but unique in terms of genetic composition.

## Materials and Methods

### Sampling and DNA Extraction

**FIGURE 1:**
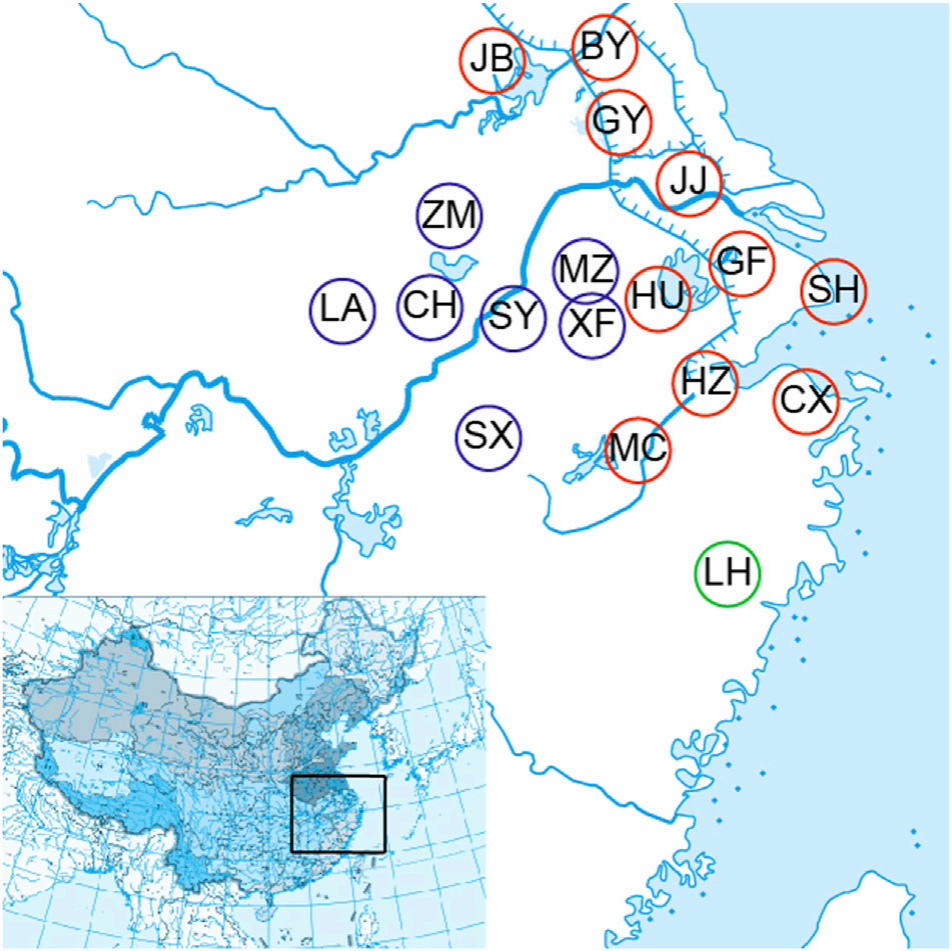
Eighteen sample collecting sites of the *Odontobutis potamophilus* including Meicheng (MC), Cixi (Cx), Hangzhou (Hz), Huzhou (HU), Baoying (by), Gaoyou (Gy), Jingjiang (JJ), Guangfu (GF), Jiangba (JB), Shanghai (SH), Meizhu (MZ), Xingfu (XF), Chaohu (CH), Shuyang (SY), Zhongmiao (ZM), Liu (LA), Shexian (SX), and Linhai (LH).

A total of 72 *O. potamophilus* were collected from Meicheng (MC), Cixi (CX), Hangzhou (HZ), Huzhou (HU), Baoying (BY), Gaoyou (GY), Jingjiang (JJ), Guangfu (GF), Jiangba (JB), Shanghai (SH), Meizhu (MZ), Xingfu (XF), Shuiyang (SY), Chaohu (CH), Zhongmiao (ZM), Luan (LA), Shexian (SX), and Linhai (LH) of the Huaihe River, the Yangtze River, the Qiantang River, and the Oujiang River basins (Table 1; Figure 1). Two samples of *O. yaluensis* were used as outgroup. Most samples were collected before 2014 when the large-scale artificial breeding and stocking started, so they are a good representation of the wild populations. The fin clips and muscle samples were taken and fixed with 75% ethanol before being brought back to the laboratory and stored at 4°C. All experimental procedures involving fish were approved by the Animal Ethics Committee of Shanghai Ocean University, China. Ezup Column Animal Genomic DNA Purification Kit (Sangon, Shanghai, China) was used to extract DNA from the tissue samples. The concentration of purified DNA was determined by a NanoDrop 3300 Fluorospectrometer (Thermo Fisher Scientific. Wilmington, DE, United States) and visualized using agarose gel electrophoresis.

**TABLE 1:** Sampling sites and date of collection.

Code	Sample ID	No. of samples	Water body	Collection site	Date
MZ	CL2	3	Lake Nanyi, Yangtze River	Meizhu,Langxi, Anhui, China	2008
XF	CL3	2	Lake Nanyi, Yangtze River	Xingfu, Langxi, Anhui	2009
HU	CL364	5	Lake Tai, Yangtze River	Huzhou, Zhejiang, China	2013/4/7
SY	CL378	3	Shuiyangjiang, Yangtze River	Shuiyang, Xuancheng, Anhui, China	2013/4/7
CH	CL382	6	Lake Caohu, Yangtze River	Chaohu, Anhui, China	2013/4/10
ZM	CL384	3	Lake Caohu, Yangtze River	Zhongmiao,Hefei, Anhui, China	2013/4/10
LA	CL399	2	Huaihe River	Liuan, Anhui, China	2013/4/10
BY	CL411	4	Lake Baoying, Yangtze River	Baoying, jiangsu, China	2013/4/10
GY	CL421	4	Lake Gaoyou, Yangtze River	Gaoyou, Jiangsu, China	2013/4/10
JJ	CL423	5	Yangtze River	Jingjiang, Jiangsu, China	2013/4/12
GF	CL478	6	Lake Tai, Yangtze River	Guangfu, Jiangsu, China	2013/6/5
JB	CL482	2	Lake Hongze,	Jiangsu, China	2013/4/19
			Huaihe River		
SH	CL495	4	Yangtze River	Shanghai, China	2013/07/04
SX	CL541	5	Yangtze River	Shexian, Huangshan, Anhui, China	2014/04/13
LH	CL763	5	Oujiang River	Linhai, Zhejiang, China	2014/6/26
MC	CL1221	5	Qiantang River	Meicheng, Zhejiang, China	2016/8/22
CX	CL1981	4	Qiantang River	Cixi, Ningbo, Zhejiang, China	2017/10/10
HZ	CL1982	4	Qiantang River	Hangzhou, Zhejiang, China	2017/10/12
Out group	CL850	1	Aihe, The Yalu River	Liaoning, China	2016/06/14
	CL1275	1	The Yalu River	Kuandian, Dandong, China	2016/11/12

### Library Construction, Gene Capture, and Sequencing

DNA was fragmented to ∼250 bp using a Covaris M220 focused ultrasonicator (Woburn, Massachusetts, United States). One µl of sheared DNA was used in agarose gel electrophoreses to check the size of sheared DNA fragments. The Illumina sequencing libraries were constructed using a modified library preparation method (Meyer and Kircher, 2010; Li et al., 2013). In the ligation step of library preparation, each sample was labeled by adding inline indices to reduce the potential risk of cross-contamination between samples in the subsequent gene capture steps. Inline indices are short 6 bp nucleotide fragments that are connected to the ends of Illumina sequencing primers. The DNA libraries were mixed equimolarly for the subsequent gene capture experiments.

According to the 4,434 single-copy nucleotide coding sequence markers of bony fish developed by Jiang et al. (2019), the RNA baits were redesigned and the area with extreme high read depth in the preliminary experiment was masked. These biotin-labeled RNA baits were synthesized based on the sequence of a freshwater goby, *Rhinogobius giurinus*. The baits’ length was 80 bp, covering the target sequences with 2X tiling. MYbaits RNA baits targeting the 4,434 loci were synthesized at Arbor Biosciences (Mycroarray, Ann Arbor, Michigan; cat#, 150901-Li-Goby).

Gene capture procedures were repeated twice followed Li et al. (2013). Briefly, sample libraries and baits were hybridized, and then the non-target fragments were washed off. The enriched libraries were amplified using IS4 and a P7 primer with an 8 bp DNA index following Meyer and Kircher (2010). After gene capture, the products were subjected to agarose gel electrophoreses for size selection, and products between 250 bp and 1,000 bp were cut and extracted. Gel extractions were sent to GENWIZ (Shanghai) for sequencing using an Illumina HiSeq platform.

### Read Assembly

The raw Illumina data were distinguished by the 8 bp P7 barcodes and the 6 bp inline indices (Yuan et al., 2016). Trim_galore v0.4.1 (http://www.bioinformatics.babraham.ac.uk/projects/trim_galore/, accessed on 10 July 2021) and cutadapt v1.2.1 (Martin, 2011) was used with default parameters to trim the adaptor sequence and reads with low-quality scores (Q < 20). The data assembly process was followed by Yuan et al. (2016). The paralogous sequences were filtered according to Yuan et al. (2016), and only loci captured for more than 30% of all individuals were kept for subsequent analyses. Clustal Omega was used to batch align the DNA sequence based on amino acid codes (Sievers et al., 2011).

### SNP Calling and Population Genetic Metrics

After aligning the assembled results and removing low-quality sequences, pick_taxa.pl was used to construct consensus sequences as a reference for SNP calling, and SNPs from coding regions were targeted (Yuan et al., 2016). The cleaned reads were mapped to consensus sequences using bwa v0.7.16a-r1181 (Li and Durbin, 2009). Samtools were used to convert the same files into binary BAM files (Li et al., 2009). Picard (http://broadinstitute.github.io/picard) was used to remove PCR repeats. GATK-3.4.0 (McKenna et al., 2010) was used to genotype the SNP loci. Thirty individuals of SH, JB, GY, BY, JJ, HU, and GF were selected for the Hardy Weinberg equilibrium test using VCFtools (Danecek et al., 2011), because our preliminary analysis suggested those would be a panmictic population. Loci that did not conform to Hardy Weinberg equilibrium were identified and removed from VCF files generated for all individuals (72 samples). SNPs were screened with the parameters including MAF> 0.05, max-missing set as 0.90, and min-meANDP set as 6 using VCFtools. Only one SNP with the least missing data and the highest quality score was retained for each locus to meet the linkage balance requirements of subsequent analyses. The optimal SNP sites of the populations were selected, and the mean pairwise population differentiation (*F*_*ST*_), nucleotide diversity (pi), and mean genetic distance (Phi_st) were calculated in the Stacks program (Catchen et al., 2013). According to the *F*_*ST*_ values between the two populations, the existence and degree of differentiation between the two populations were inferred (Wright, 1968).

### Population Clustering and Genetic Structure

The captured genes were concatenated for phylogenetic tree inference. The phylogenetic tree was constructed using the maximum likelihood method to reveal the relationship of 72 *O. potamophilus* with two *O. yaluensis* as outgroups. The ML tree was reconstructed using RAxMLv8.0.0 under the GTRGAMMA model with 1000 bootstraps (Stamatakis, 2014). Figtree v1.4.2 was used to visualize the results (http://tree.bio.ed.ac.uk/software/Figuretree/).

STRUCTURE v2.3.4 (Pritchard et al., 2000) was used to evaluate the population structure of the 72 individuals of *O. potamophilus*. The individuals were coded as 18 populations according to their geographical sampling sites. The STRUCTURE operating parameter was set to 20,000 for the length of the burn-in period, and 200,000 for the number of MCMC reps after burn-in. The analyses of k = 1–18 were repeated five times. The most likely K value was determined using Structure Harvester 0.6.93 (Earl, 2012).

The discriminant analysis of principal components (DAPC) was performed twice for each replication using R (Team, 2013) package adegenet version 2.1.1 (Jombart, 2008). The first time was used to evaluate the optimal number of PCs, and this value was used for the second analysis. The 72 individuals were divided into 18 groups by sampling sites for the cluster parameters. According to the number of PC lowest RMSE, the number of PCs chosen (≥1) was 10, and the number of distinct functions (≥1) was three. The scattering parameter was used for drawing.

### History of Population Divergence

The results of cluster analysis showed that fish from the 18 sampling sites could be lumped into three major groups: The Oujiang clade (OJ), the Qiantang and lower Yangtze clade (QY), and the middle Yangtze clade (MY). We hypothesized four models for the evolutionary history of the three groups: model 1–3, either OJ, QY, or MY diverged first from the other two groups; model 4, the best model from the previous three but with migration allowed between different groups.

Different models were tested using fastsimcoal2 version 2.7 (Excoffier, 2011; Excoffier and Foll, 2011; Excoffier et al., 2021). Fastsimcoal2 can infer the population history by simulating historical events; it also can use the joint site frequency spectrum (JSFS) between populations as a summary statistics. Parameters include time, source, sink, migrants, new size, new growth rate, and migration matrix. The VCF file was converted into *.arp file using PGDSpider 2.1.1.5 (Lischer and Excoffier, 2012). The result of the Arp file was used as the input file for Alequin3.5 (Excoffier and Lischer, 2006) to calculate the site frequency spectrum (SFS). Based on the calculated SFS file (*.obs), different evolution scenarios were simulated in Fastsimcoal 2.7. Each model runs 100 independent point estimates, including 100 K coalescent simulation, and 30 ECM cycles (“-L 30”). The optimal JSFS observed by the model was compared with the Akaike information criterion (AIC). The conditional interval (CI) of the optimal model’s point estimation was performed at 10 runs per bootstrapped SFS.

## Results

### Next-Generation Sequencing Data

A total of 287,518,950 reads were generated, with an average of 3,993,319 reads per individual. A total of 223,207,564 reads were retained, after the adapter sequences and the low-quality sequence (Q < 20) were trimmed, with an average of 3,100,106 reads per individual. After excluding PCR duplicates, 77.63% of the trimmed data were retained. An average of 2,635 loci were enriched in each sample, with the highest of 3,571 loci captured in CL1221_2, and the lowest of 202 loci in CL 1981_5. There were 3,750 loci on average captured for the two outgroup samples (Supplementary Table S1). After manual examination of all loci of *O. potamophilus* and *O. yaluensis*, 4,267 genes (96.23% of 4434 genes) were enriched in more than 22 (>30%) individuals and used for phylogenetic reconstruction.

### Genetic Diversity and Differentiation

There were 12,988 SNPs called in total, with an average of 4.9 SNPs per locus. There were 6,045 out of 7,105 variants passing filters and HWE test in the 30 individuals examined. Those 1,060 sites that did not pass the HWE test were removed from the variants in the whole dataset (72 samples), keeping 11,932 out of the 12,988 SNPs. At least one SNP was called for 472 loci after selecting one SNP from each locus to avoid linkage disequilibrium. The average nucleotide diversity (pi) of variant positions was 0.1250 with the highest 0.1846 for MC, and the lowest 0.0471 found in LH (Table 2). Populations with large genetic difference (*F*_*ST*_ > 0.25) accounted for 60.13%, medium degree of genetic difference (0.25 > *F*_*ST*_ > 0.15) accounted for 16.34%, and existed genetic difference (0.05 < *F*_*ST*_ < 0.15) accounted for 23.53%. LH, ZM, LA, and SX were the most divergent populations from the others. The maximum genetic divergence was found between LH and SX (*F*_*ST*_ = 0.7077), and the lowest genetic differentiation was between GF and HU (*F*_*ST*_ = 0.0492) (Table 3).

**TABLE 2:** The nucleotide diversity (pi) of *Odontobutis potamophilus* of each population, including variant positions and all positions.

Group	Code	pi (variant positions)
QY	MC	0.1846
	CX	0.1289
	HZ	0.1391
	HU	0.1673
	BY	0.1374
	GY	0.1438
	JJ	0.1365
	GF	0.1461
	JB	0.1188
	SH	0.1562
MY	MZ	0.1119
	XF	0.1335
	CH	0.1330
	SY	0.1351
	ZM	0.1114
	LA	0.0487
	SX	0.0701
OJ	LH	0.0471

**TABLE 3:** Pairwise differences (*F*_*ST*_) among populations.

	MZ	XF	SY	LA	CH	ZM	SX	MC	CX	GF	HZ	GY	JJ	JB	BY	SH	HU
LH	0.6355	0.6377	0.5929	0.7501	0.5501	0.6591	0.7077	0.4404	0.5925	0.4839	0.5538	0.5652	0.5585	0.6907	0.5679	0.5380	0.4869
MZ		0.1309	0.1332	0.3709	0.1215	0.2005	0.2901	0.2583	0.3634	0.2770	0.3293	0.3418	0.3251	0.4451	0.3376	0.3198	0.2747
XF			0.1211	0.3585	0.1024	0.1802	0.2988	0.2441	0.3700	0.2729	0.3362	0.3458	0.3307	0.4562	0.3477	0.3163	0.2740
SY				0.2889	0.0926	0.1533	0.2062	0.2476	0.3387	0.2758	0.3222	0.3285	0.3163	0.4012	0.3243	0.3108	0.2725
LA					0.2080	0.3546	0.4373	0.2500	0.4330	0.2951	0.3822	0.3968	0.3730	0.5644	0.3989	0.3592	0.3002
CH						0.0805	0.1930	0.2476	0.3071	0.2657	0.2946	0.3064	0.2963	0.3513	0.3073	0.2926	0.2621
ZM							0.2884	0.2765	0.3856	0.3004	0.3607	0.3725	0.3539	0.4686	0.3672	0.3477	0.3067
SX								0.3452	0.4576	0.3677	0.4271	0.4361	0.4177	0.5311	0.4266	0.4173	0.3786
MC									0.1814	0.1579	0.1860	0.2033	0.1970	0.2075	0.2048	0.1809	0.1568
CX										0.1204	0.1758	0.1672	0.1615	0.2298	0.1704	0.1541	0.1283
GF											0.0829	0.0866	0.0829	0.1039	0.0887	0.0712	0.0492
HZ												0.1419	0.1305	0.1906	0.1382	0.1229	0.0827
GY													0.0821	0.1195	0.0748	0.1027	0.0840
JJ														0.1213	0.0823	0.1136	0.0880
JB															0.1253	0.1329	0.1047
BY																0.1133	0.0913
SH																	0.0679

### Population Structure

**FIGURE 2:**
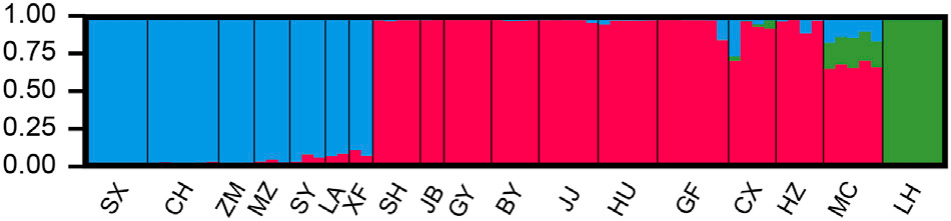
The results of STUCTURE analysis of *Odontobutis potamophilus* populations based on 472 SNP loci. Red denotes the Qiantang and lower Yangtze group (QY), blue denotes the middle Yangtze group (MY), and green denotes the Oujiang clade (OJ). Each individual is represented by a single bar. The different color on a single bar indicates admixture origin of the individual.

**FIGURE 3:**
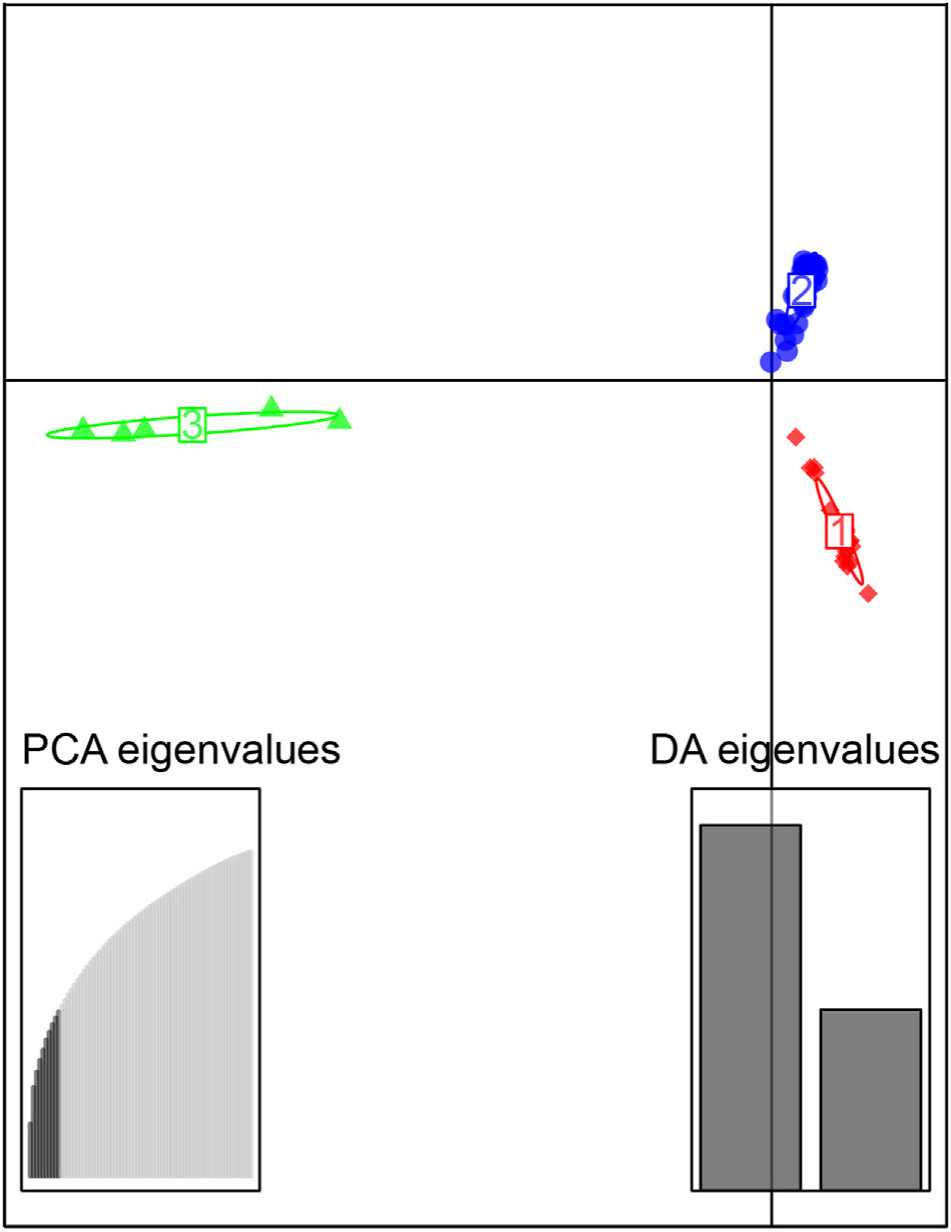
Scatterplot generated by the discriminant analysis of principal components (DAPC). The 72 individuals were divided into 3 clusters, red, the Qiantang and lower Yangtze group (QY), blue, the middle Yangtze group (MY), and green, the Oujiang group (OJ).

**FIGURE 4:**
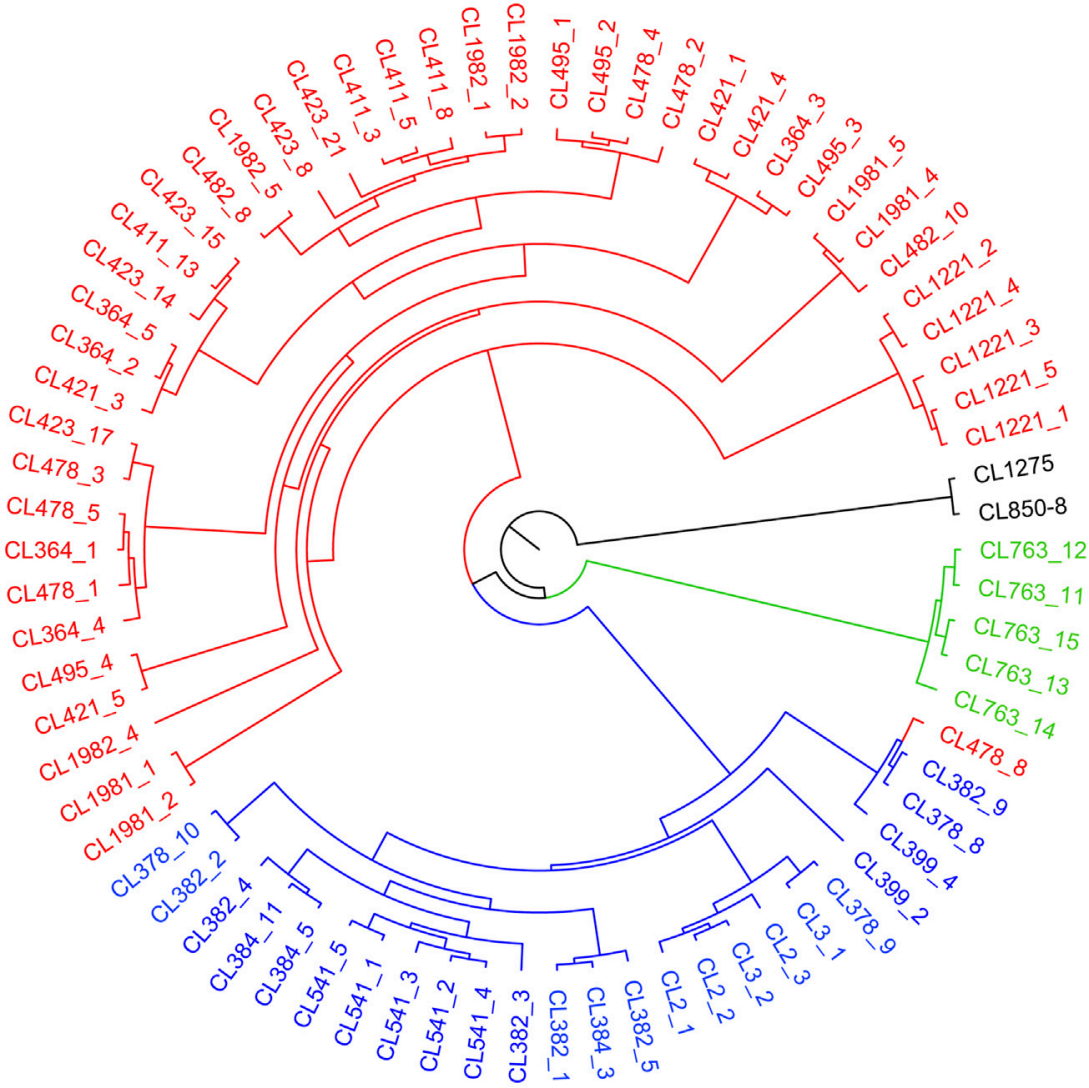
A maximum likelihood tree based on captured genes using RAxML under GTRGAMMAR mode with 1,000 bootstrap replicates. The tree consists four parts, including red QY clade (the Qiantang and lower Yangtze clade), blue MY clade (the middle Yangtze clade), green OJ clade (the Oujiang clade), and black outgroup clade.

The sampled sites can be divided into three groups (k = 3) (Supplementary Figure S1), including the Oujiang group (OJ, green), the Qiantang and lower Yangtze group (QY, red), and the middle Yangtze group (MY, blue) (Supplementary Table S2; Figure 2). Among the three groups, MC and CX populations had the most admixture individuals. These individuals were dominated by red loci, and green and blue loci only accounted for a small part. LH, GY, JB, and SX populations were highly independent, and they had almost no admixture with the other groups. Results of DAPC also showed that the populations of *O. potamophilus* can be divided into three groups (Figure 3). According to the results of the ML tree (Figure 4), the first clade split off was the Oujiang clade (OJ, green), and then the Qiantang and lower Yangtze clade (QY, red), and the middle Yangtze clade (MY, blue). This pattern cooperated with the results of STRUCTURE analysis and DAPC.

### Historical Population Dynamics of *O. potamophilus*

**FIGURE 5:**
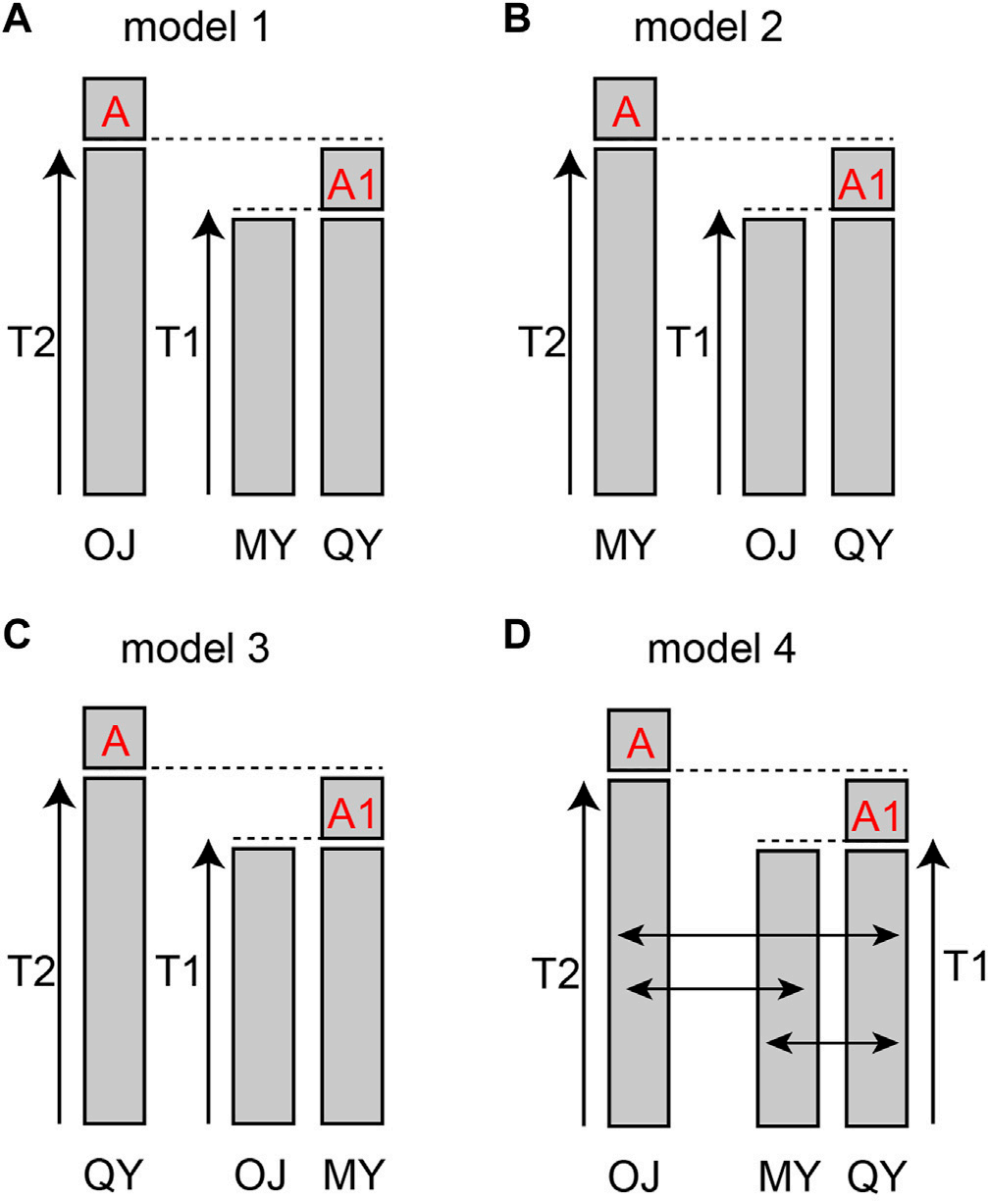
Models of population history of *Odontobutis potamophilus*. **(A)** OJ split off first, and the other two groups differentiated later from a common ancestor A_0_. **(B)** the first population diverged was MY. **(C)** the first population split off was QY. **(D)** the gene flow parameters added to model 1.

**FIGURE 6:**
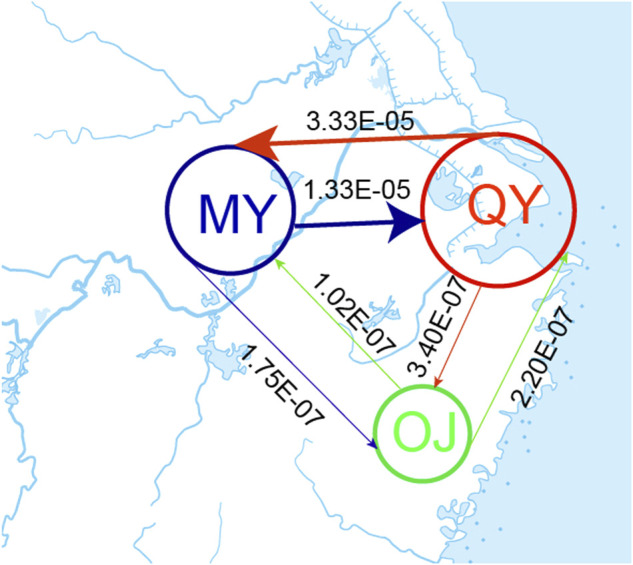
Estimated gene flow between adjacent clades. The number on the line showed the strength of gene flow. The Oujiang clade (OJ), the Qiantang and lower Yangtze clade (QY), and the middle Yangtze clade (MY).

The bestlhoods file generated using fastsimcoal2 analyses has two types of likelihoods, including MaxObsLhood and MaxEstLhood. MaxObsLhood is the maximum possible value for the expected SFS to match the observed SFS. MaxEstLhood is the maximum likelihood estimated according to model parameters. The higher the model fit with the population history, the smaller the difference between MaxObsLhood and MaxEstLhood. The MaxObsLhood values of all four models were −155.92 (Table 4). Model 1 had the lowest MaxEstLhood (−222.96) while model 2 had the largest (−228.8) (Supplementary Figure S2). Therefore, the most suitable model among the first three models was model 1. Based on the genetic structure of model 1, the parameters of gene flow were added to formulate model 4. Model 4 had smaller a MaxObsLhood (−217.9) than model 1 (Table 4), and Model 4 also had the lowest AIC value (1013.46), so we chose model 4 as the best model to explain the population history of *O. potamophilus* (Table 4). According to model 4, the common ancestor A_0_ differentiated into OJ and A_1_, and then A_1_ differentiated into MY and QY clades (Figure 5). There was gene flow among all three clades, but MY and QY clades had higher gene flow. The gene flow between MY and QY clades was 100-fold larger than the gene flow between OJ and QY (or MY) (Figure 6).

**TABLE 4:** Comparing different models of population divergence history of *Odontobutis potamophilus* based on site frequency spectrum (SFS).

	MaxEstLhood	MaxObsLhood	AIC	Delta-likelihood
Modle1	−222.96	−155.92	1036.79	67.05
Modle2	−228.80	−155.92	1063.68	72.89
Modle3	−226.98	−155.92	1055.28	71.06
Modle4	−217.90	−155.92	1013.46	61.98

## Discussions

### Genetic Diversity and Candidate Populations for Conservation

Genetic diversity plays an important role in the survival and adaptability of species and it is also considered a basis for genetic breeding and conservation (Frankham, 2005; King and Lively, 2012). This study provides a framework for the conservation of *O. potamophilus*. The higher the genetic diversity of the populations, the stronger the adaptability to environmental changes. Therefore, populations with high genetic diversity are more suitable as founder population for a breeding program to be protected. The principle of selecting parents is to select populations with high genetic diversity and no admixed individuals in each group as ideal parents. The higher the genetic diversity, the richer the genetic information carried by the species, which is conducive to the subsequent breeding of excellent varieties. On the basis of high genetic diversity, we selected populations from each group without hybridized individuals from the other two groups, so as to avoid carrying the same genetic information between the two populations as parents. Thus, maintaining and improving the good quality of the progenies of *O. potamophilus*.

In the OJ group, LH was the only population sampled, with a nucleotide diversity of 0.0471. Mean pairwise population differentiation (*F*_*ST*_) is an indicator to measure genetic differences between populations. The maximum value of *F*_*ST*_ was 0.7077 between LH and SX, indicating the greatest genetic difference between the two populations. In fact, the *F*_*ST*_ values between LH and all other populations were greater than 0.25, suggesting that LH is a unique genetic resource and breeding material. Therefore, LH should be protected for breeding programs, but more sites in the Oujiang River basin should be surveyed to examine the genetic diversity of *O. potamophilus* within the Oujiang River system. In the MY group, the population with the highest nucleotide diversity was SY (pi = 0.1351). However, many admixed individuals were observed in the SY population, so we should not use it for breeding programs. We suggest that the CH population, which had the second high nucleotide diversity (pi = 0.1330) but with no admixed individuals could be used for breeding projects. In the QY group, the population with the highest nucleotide diversity was MC (pi = 0.1846) but many admixed individuals were found in this population, so we suggest the GY (pi = 0.1438) population as the representative population due to the higher pi value and no admixed individuals. Taking the parameters of nucleotide diversity, *F*_*ST*_, and admixture status all into consideration, we suggest that LH, GY, and CH populations should be protected. Those populations could be the preferred resource as founder populations for breeding projects, representing the OJ, QY, and MY groups, respectively.

Conservation is urgently needed in some populations, such as LA (Pi = 0.0487) and SX (Pi = 0.0701), due to their low genetic diversity compared to other populations. In these populations with low genetic diversity, the loss of individuals may lead to a decrease in the ability of the population to adapt to long-term environmental changes. Therefore, populations with low genetic diversity also should be protected, for example, by banning illegal fishing, to prevent anthropogenic destruction of the ecological environment, and creating a favorable habitat environment to maintain the wild populations of *O. potamophilus*.

### Population Structure and History

*Odontobutis potamophilus* is distributed in the Huaihe River, the Yangtze River, the Qiantang River, and the Oujiang River basins; however, how many divergent groups of *O. potamophilus* in these river systems have not been identified previously. The results of ML tree, DAPC, and STRUCTURE analyses unequivocally supported the division of *O. potamophilus* into three groups. The first differentiated group (OJ) was a clade of samples collected from the Oujiang River at Linhai, Zhejiang Province. Compared with other water systems, the Oujiang River is located in the southernmost part of the distribution range, so OJ population can be regarded as an independent population and it is crucial to protect the population of *O. potamophilus* in Oujiang. The other populations were divided into two groups, with Lake Tai as the boundary. The QY group is located to the east of Lake Tai, which is composed of populations from the Huaihe River, the Qiantang River, and lower Yangtze River. To the west of Lake Tai is MY group, which is composed of populations from the middle Yangtze River in Anhui Province. A similar pattern was also reported in previous research (Li, 2015). Most lakes in the middle and lower reaches of the Yangtze River dried up during the glacial periods due to the decline of sea level (Yang et al., 2000). The decline of sea level might lead to the isolation of the QY group in coastal shelters from the MY group. Lake Tai formed at about 3.7 Ka after the rise of sea level (Hong, 1991). During this period, Lake Tai might become a hybrid zone of QY and MY. The patterns of species differentiation and hybridization around Lake Tai also were found in other fishes, such as *Coilia nasus* (Cheng et al., 2019).

STRUCTURE results revealed that many populations of MY and QY had gene flow between groups. The geographical locations of these two groups are different sections of the Yangtze River. After the sea level rise, the previously isolated lakes were connected to the Yangtze. Therefore, individuals with admixed alleles of MY and QY were found in populations around Lake Tai, such as SY, XF, and GF. The individuals of LH (OJ group) had little gene flow with QY groups, probably because the Oujiang River has no direct connections with the Qiantang River or the Yangtze River. There were possibly few river capture events between the Oujiang River and the Qiantang River, therefore, a few individuals from MC and CX (the Qiantang River) showed admixture genotype with some alleles from the LH population (Figure 2).

Sample CL478-8 was collected from GF, which belongs to the QY group but clustered with individuals of the MY group in the phylogenetic tree (Figure 4). We speculate that CL478-8 was a hybrid between QY and MY. Moreover, the result of population history dynamics is consistent with the STRUCTURE results, that gene flow between MY and QY was the highest. We suggested that *O. potamophilus* should be divided into three management units, that is, the group to the east of Lake Tai, the group to the west of Lake Tai, and the Oujiang group.

### Perspectives on Selective Breeding of *O. potamophilus*

If the volume of farmed *O. potamophilus* can meet consumer demand, the quantities of wild *O. potamophilus* caught can be reduced. Although artificial breeding of *O. potamophilus* has been successful, there is yet no selected strain for aquaculture. Nonetheless, within-species crossing produced obvious heterosis in *O. potamophilus* (Wang et al., 2016), so there is high potential for selective breeding of *O. potamophilus*. A famous example of breeding based on within-species crossing and subsequent selection is the “GIFT tilapia”. The successful breeding of GIFT tilapia indicates that the high genetic diversity of founder populations can improve the genetic gain of breeding (Ponzoni et al., 2005). We argue that a similar strategy can be applied to the selective breeding of *O. potamophilus*, that is, a founder population can be formulated by crossing LH, GY, and CH, the representative populations of the three groups to increase the background diversity of the founder population; and, then subsequent selection can be carried out on it. Meanwhile, the wild populations of the three groups should be conserved separately to keep the wild diversity of this fish.

## Conclusion

In this study, we found that *O. potamophilus* could be divided into three major groups. Based on the nucleotide diversity, pairwise population differentiation, and admixture status of the populations analyzed, we recommended three candidates for the founder population and a strategy for selective breeding for *O. potamophilus*. All of the *O. potamophilus* populations should be considered for conservation to preserve their genetic diversity.

## Data Availability

The datasets presented in this study can be found in online repositories. The names of the repository/repositories and accession number(s) can be found below: https://www.ncbi.nlm.nih.gov/, PRJNA792327.
