# Supplementary material for: Population Structure, Genetic Diversity, and Conservation Strategies of a Commercially Important Sleeper Fish, Odontobutis potamophilus (Gobiiformes: Odontobutidae) Based on Gene-Capture Data
Source: Front Genet. 2022 May 24;13:843848. doi: 10.3389/fgene.2022.843848 (PMC9171042; doi:10.3389/fgene.2022.843848)

**Supplementary materials:**

**Table S1.** The sequencing results of 72 individuals of *Odontobutis potamophilus* and the two outgroup, *Odontobutis sinensis*.

**Table S2.** The 72 individuals of *Odontobutis potamophilus* were divided into three groups according to the STRUCTURE analysis.

**Figure S1.** Optimal Delta K value for STRUCTURE statistics of the 72 individuals of *Odontobutis potamophilus*.

**Figure S2.** Optimal Likelihood of population history models

**Table S1** The sequencing results of 72 individuals of *Odontobutis potamophilus* and the two outgroup, *Odontobutis sinensis*.

| Sample id | Num. of captured loci (%) | Num. of trimmed reads | Num. of trimmed bases (bp) | Num. of deduplicated reads | Num. of deduplicated bases (bp) |
| --- | --- | --- | --- | --- | --- |
| CL1222-1 | 3,060 (69%) | 1,979,928 | 283,748,392 | 1,703,624 | 243,089,472 |
| CL1221_2 | 3,571 (81%) | 6,943,464 | 999,858,816 | 5,905,722 | 850,423,968 |
| CL1221_3 | 3,088 (70%) | 1,966,120 | 283,121,280 | 1,716,598 | 247,190,112 |
| CL1221_4 | 2,888 (65%) | 1,702,830 | 243,968,918 | 1,442,186 | 205,923,760 |
| CL1221_5 | 2,446 (55%) | 850,268 | 121,724,334 | 732,716 | 104,496,696 |
| CL1981_1 | 2,849 (64%) | 13,121,542 | 1,873,877,194 | 11,000,950 | 1,565,468,779 |
| CL1981_2 | 1,419 (32%) | 5,236,058 | 749,542,776 | 4,388,484 | 626,728,996 |
| CL1981_4 | 1,398 (32%) | 1,666,858 | 238,536,926 | 1,359,194 | 194,043,952 |
| CL1981_5 | 202 5% () | 1,798,322 | 257,234,808 | 1,470,948 | 209,772,813 |
| CL1982_1 | 1,834 41% () | 6,250,858 | 893,696,170 | 5,259,304 | 749,557,463 |
| CL1982_2 | 2,939 (66%) | 5,702,322 | 814,544,004 | 4,584,844 | 652,784,558 |
| CL1982_4 | 1,927 (43%) | 4,168,584 | 595,858,344 | 3,346,140 | 476,519,512 |
| CL1982_5 | 1,151 (26%) | 2,769,790 | 396,275,120 | 2,306,184 | 328,930,489 |
| CL2-1 | 2,351 (53%) | 3,802,086 | 543,762,936 | 2,856,544 | 407,619,064 |
| CL2-2 | 2,553 (58%) | 2,681,330 | 383,248,572 | 1,983,458 | 282,614,178 |
| CL2-3 | 1,083 (24%) | 716,630 | 102,349,458 | 535,660 | 76,336,314 |
| CL364-1 | 2,599 (59%) | 690,598 | 98,811,406 | 580,746 | 82,825,744 |
| CL364-2 | 3,283 (74%) | 2,600,138 | 372,645,498 | 2,120,632 | 302,794,423 |
| CL364-3 | 3,316 (75%) | 3,660,208 | 523,714,756 | 2,926,806 | 416,987,483 |
| CL364-4 | 2,598 (59%) | 1,272,632 | 182,134,874 | 1,088,912 | 155,344,731 |
| CL364-5 | 3,216 (73%) | 4,592,946 | 656,554,240 | 3,694,606 | 526,954,201 |
| CL378-8 | 3,648 (82%) | 11,142,658 | 1,660,566,498 | 6,779,138 | 1,004,515,433 |
| CL378-9 | 1,742 (39%) | 290,276 | 41,480,716 | 246,880 | 35,099,175 |
| CL378-10 | 3,344 (75%) | 6,618,474 | 945,357,290 | 5,033,450 | 716,598,644 |
| CL382-1 | 3,252 (73%) | 3,380,284 | 483,797,978 | 2,748,954 | 391,009,605 |
| CL382-2 | 1,420 (32%) | 1,372,186 | 196,061,708 | 967,846 | 137,034,375 |
| CL382-3 | 2,960 (67%) | 4,013,012 | 574,514,226 | 2,827,572 | 402,109,487 |
| CL382-4 | 3,488 (79%) | 5,045,586 | 722,430,084 | 4,056,324 | 578,333,909 |
| CL382-5 | 716 (16%) | 6,436,770 | 918,912,442 | 4,568,756 | 649,177,224 |
| CL382-9 | 3,208 (72%) | 3,614,862 | 538,230,482 | 2,034,016 | 299,121,843 |
| CL384-3 | 2,714 (61%) | 3,138,200 | 448,301,592 | 2,466,854 | 350,889,864 |
| CL384-5 | 1,385 (31%) | 1,512,584 | 215,630,128 | 1,027,690 | 145,715,199 |
| CL384-11 | 3,494 (79%) | 4,134,186 | 591,664,248 | 3,178,892 | 453,608,289 |
| CL399-2 | 3,561 (80%) | 7,953,844 | 1,138,120,644 | 5,901,028 | 840,520,908 |
| CL399-4 | 3,197 (72%) | 10,479,760 | 1,556,948,676 | 7,150,158 | 1,038,956,679 |
| CL3-1 | 2,814 (64%) | 3,755,254 | 537,096,526 | 2,987,852 | 425,689,071 |
| CL3-2 | 3,300 (74%) | 3,061,498 | 438,302,128 | 2,538,886 | 362,208,123 |
| CL411-3 | 1,925 (43%) | 955,814 | 136,578,336 | 722,956 | 102,938,616 |
| CL411-5 | 2,113 (48%) | 1,323,652 | 188,775,112 | 1,004,200 | 142,715,720 |
| CL411-8 | 2,957 (67%) | 2,308,988 | 329,597,114 | 1,836,108 | 261,098,323 |
| CL411-13 | 2,640 (60%) | 2,589,546 | 370,497,454 | 2,108,472 | 301,080,806 |
| CL421-1 | 3,289 (74%) | 4,075,804 | 583,379,638 | 3,374,932 | 480,819,208 |
| CL421-3 | 3,631 (82%) | 10,943,552 | 1,628,377,912 | 8,182,220 | 1,203,550,656 |
| CL421-4 | 3,368 (76%) | 5,700,696 | 816,266,470 | 4,894,186 | 698,373,756 |
| CL421-5 | 2,467 (56%) | 872,098 | 124,721,630 | 747,520 | 106,528,101 |
| CL423-8 | 2,601 (59%) | 4,196,776 | 599,667,244 | 3,513,572 | 500,894,417 |
| CL423-14 | 1,859 (42%) | 4,522,880 | 645,898,262 | 3,764,592 | 535,068,253 |
| CL423-15 | 3,440 (78%) | 6,344,488 | 908,548,478 | 5,103,604 | 729,062,590 |
| CL423-17 | 1,875 (42%) | 12,244,456 | 1,746,175,970 | 8,092,454 | 1,149,385,675 |
| CL423-21 | 712 (16%) | 3,564,028 | 506,754,832 | 2,491,992 | 351,899,994 |
| CL478-1 | 2,267 (51%) | 1,250,224 | 178,715,784 | 985,646 | 140,060,376 |
| CL478-2 | 3,139 (71%) | 2,661,592 | 381,079,142 | 2,054,306 | 292,581,240 |
| CL478-3 | 2,031 (46%) | 1,240,116 | 177,162,128 | 1,043,394 | 148,262,547 |
| CL478-4 | 3,339 (75%) | 10,574,542 | 1,512,012,986 | 8,232,710 | 1,168,467,226 |
| CL478-5 | 2,664 (60%) | 4,112,950 | 586,465,412 | 3,417,452 | 485,619,054 |
| CL478-8 | 3,029 (68%) | 2,680,736 | 398,985,392 | 1,393,960 | 204,833,156 |
| CL482-8 | 2,224 (50%) | 664,278 | 95,051,392 | 487,532 | 69,361,401 |
| CL482-10 | 1,082 (24%) | 2,279,052 | 325,802,362 | 1,561,586 | 222,361,999 |
| CL495-1 | 3,470 (78%) | 9,103,388 | 1,302,925,130 | 7,251,386 | 1,030,618,099 |
| CL495-2 | 3,284 (74%) | 3,493,238 | 500,406,352 | 2,912,474 | 415,187,649 |
| CL495-3 | 3,316 (75%) | 5,017,540 | 718,253,872 | 4,166,876 | 594,402,317 |
| CL495-4 | 3,411 (77%) | 4,313,128 | 617,784,098 | 3,709,180 | 529,275,844 |
| CL541-1 | 3,543 (80%) | 8,696,322 | 1,244,352,832 | 6,759,304 | 961,138,106 |
| CL541-2 | 3,090 (70%) | 2,714,024 | 388,115,494 | 2,205,822 | 313,748,070 |
| CL541-3 | 2,957 (67%) | 2,219,844 | 317,614,572 | 1,846,402 | 262,652,438 |
| CL541-4 | 3,210 (72%) | 3,612,342 | 516,925,110 | 2,860,958 | 407,177,754 |
| CL541-5 | 3,172 (72%) | 2,248,050 | 321,688,036 | 1,800,136 | 256,585,306 |
| CL763-11 | 3,562 (80%) | 5,373,210 | 769,826,676 | 4,519,762 | 645,990,988 |
| CL763-12 | 2,867 (65%) | 1,388,854 | 198,759,178 | 1,168,834 | 166,571,388 |
| CL763-13 | 1,754 (40%) | 477,850 | 68,072,326 | 403,426 | 57,240,878 |
| CL763-14 | 3,137 (71%) | 3,236,722 | 463,310,902 | 2,740,998 | 390,619,902 |
| CL763-15 | 2,227 (50%) | 395,224 | 56,549,886 | 330,058 | 47,063,368 |
| CL850-8 | 3,541 (80%) | 11,357,578 | 1,688,582,750 | 8,558,536 | 1,254,805,006 |
| CL1275 | 3,600 (81%) | 20,483,636 | 3,053,069,044 | 14,302,702 | 2,108,795,409 |

Table S2 The 72 individuals of *Odontobutis potamophilus* were divided into three groups according to the STRUCTURE analysis.

| Group | Population | Sample ID |
| --- | --- | --- |
| QY | MC | CL1222-1，CL1221-2，CL1221-3，CL1221-4，CL1221-5 |
|  | CX | CL1981_1，CL1981_2，CL1981_4，CL1981_5 |
|  | HZ | CL1982_1，CL1982_2，CL1982_4，CL1982_5 |
|  | HU | CL364-1，CL364-2，CL364-3，CL364-4，CL364-5 |
|  | BY | CL411-3，CL411-5，CL411-8，CL411-13 |
|  | GY | CL421-1，CL421-3，CL421-4，CL421-5 |
|  | JJ | CL423-8，CL423-14，CL423-15，CL423-17，CL423-21 |
|  | GF | CL478-1，CL478-2，CL478-3，CL478-4，CL478-5，CL478-8 |
|  | JS | CL482-8,CL482-10 |
|  | SH | CL495-1,CL495-2,CL495-3,CL495-4 |
| MY | MZ | CL2-1,CL2-2,CL2-3 |
|  | XF | CL3-1,CL3-2 |
|  | SY | CL378-8,CL378-9,CL378-10 |
|  | CH | CL382-1,CL382-2,CL382-3,CL382-4,CL382-5,CL382-9 |
|  | ZM | CL384-3,CL384-5,CL384-11 |
|  | LA | CL399-2,CL399-4 |
|  | SX | CL541-1,CL541-2,CL541-3,CL541-4,CL541-5 |
| OJ | LH | CL763-11，CL763-12，CL763-13，CL763-14，CL763-15 |

Figure S1 Optimal Delta K value for STRUCTURE statistics of the 72 individuals of *Odontobutis potamophilus*.


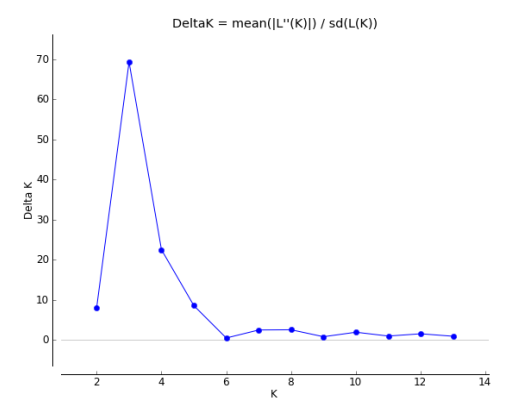


Figure S2 Optimal Likelihood of population history models.


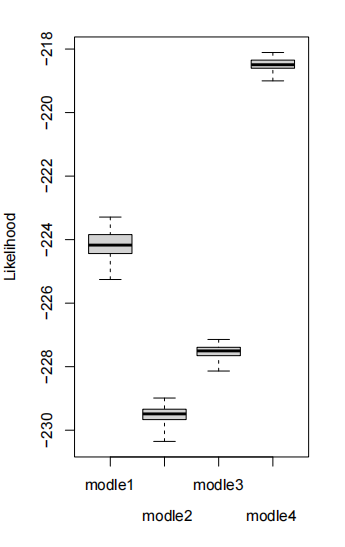

Supplement: Supplementary file 1 [file DataSheet1.docx]
